# Improved Information and Educational Messages on Outer Packaging of Micronutrient Powders Distributed in Indonesia Increase Caregiver Knowledge and Adherence to Recommended Use

**DOI:** 10.3390/nu10060747

**Published:** 2018-06-08

**Authors:** Aang Sutrisna, Marieke Vossenaar, Alia Poonawala, Agnes Mallipu, Doddy Izwardy, Ravi Menon, Alison Tumilowicz

**Affiliations:** 1 GAIN, Menara Palma 7th floor unit 705, Jl. Rasuna Said Blok X2 Kav 6, Jakarta 12950, Indonesia; asutrisna@gainhealth.org (A.S.); amallipu@gainhealth.org (A.M.); rmenon@gainhealth.org (R.M.); 2 GAIN, Rue de Varembé 7, 1202 Geneva, Switzerland; mvossenaar@gainhealth.org (M.V.); apoonawala@gainhealth.org (A.P.); 3 Directorate of Community Nutrition, Jln. Rasuna Said Blok X-5 Kav. 4-9, Jakarta 12950, Indonesia; izwardydoddy@gmail.com

**Keywords:** infants and young children, micronutrient powders, adherence, packaging, Indonesia

## Abstract

The objective of this study was to examine the influence of improved information and educational messages on outer packaging of a micronutrient powder (MNP), locally known as “*Taburia*”, on knowledge and adherence to recommended use. A community-based cluster randomized controlled trial was conducted among 1149 caregivers and their children aged 6–36 months. Caregiver–child dyads were randomized by their villages to receive 30 sachets of *Taburia* with the: (i) original outer packaging; (ii) improved outer packaging; or (iii) improved outer packaging combined with cooking demonstrations. Adherence to *Taburia* use was assessed through caregiver interviews and observation of unused sachets during home visits; “high” adherence was defined as consuming 13–17 sachets in the previous month. Data collection included surveys and focus groups discussions. The majority of caregivers (>80%) preferred the improved packaging because it was more attractive and contained more comprehensive information. Caregivers who received the improved packaging had better knowledge regarding the recommended use of *Taburia* (*p* < 0.001) and higher adherence with the prescribed use of *Taburia* (43% with “high” adherence) (*p* < 0.001) than those who received the original packaging (29% with “high” adherence). Caregivers who participated in cooking demonstrations generally had better knowledge regarding the benefits of *Taburia* and recommended use, but this did not lead to higher adherence to recommended use. “Underconsumption” of *Taburia* (≤7 sachets) was much less prevalent than “overconsumption” (≥23 sachets), and original packaging users were more likely to consume *Taburia* daily instead of every two days as recommended. We conclude that the design of the outer packaging and comprehensiveness of information provided are important influencers of recommended MNP use by caregivers.

## 1. Introduction

Indonesia has made some progress towards achieving the Millennium Development Goal target on the reduction of stunting in children under five; however, malnutrition remains widespread [1]. National estimates indicate that 37% of children under five (around eight million) are stunted and 28% suffer from anaemia [2]. Malnutrition in Indonesia is primarily attributed to high rates of disease and poor infant and young child feeding (IYCF) practices, particularly low rates of exclusive breastfeeding, low dietary diversity, inadequate consumption of iron-rich foods, the absence of responsive feeding following the principles of psycho-social care and poor hygiene practices [3,4,5]. The latest Indonesian Demographic and Health Survey (DHS) provides key World Health Organization (WHO) IYCF indicators among children 6–23 months old; it reports that 52% consume a minimum dietary diversity, 61% of children meet recommendations for minimum meal frequency, and 34% consume a minimum acceptable diet [5]. Consequently, dietary intakes of Indonesian infants and young children are not fulfilled for several micronutrients such as vitamin A, iron and zinc [6,7,8].

The “1000 days” from conception to age two is widely recognized as a window of opportunity for the prevention of malnutrition and its short and long-term consequences [9,10]. The longest stage in this window is the period of complementary feeding, which ideally starts at six months of age and continues up to two years of age, in which young children move from being exclusively breastfed to receiving nutrients from a range of foods in addition to breastmilk. Achieving nutrient adequacy during this period is challenging as young children eat in small amounts and nutrient adequacy can only be achieved through the consumption of nutrient dense foods [11,12]. Point-of-use fortification of complementary foods with iron-containing micronutrient powders (MNP) is recommended by the WHO as a strategy to improve iron status and reduce anaemia in infants and young children [13]. In 2011, the government of Indonesia initiated free distribution of MNP locally branded as *Taburia* targeting low-income households with children aged 6–24 months via community health posts (*Posyandu*) across three provinces and 64 districts. The term *Taburia* was derived from the Indonesian word “*tabur*” which means “to sprinkle” and the word “*ceria*” which means “cheerful”, thus alluding to an easy-to-use supplement that can make a child cheerful. *Taburia* distribution has faced several programmatic challenges, resulting in low acceptability of the product and inadequate adherence to prescribed use [14,15]. As previously described, caregivers observed changes in appearance and organoleptic properties of foods typically consumed by young children with the addition of *Taburia*, even in a controlled setting following preparation instructions (such as not mixing *Taburia* with hot foods). In this sensory evaluation, *Taburia* (consisting of 14 micronutrients including iron as ferrous fumarate) was found to enhance sweetness, saltiness, and umami tastes, but was also perceived as slightly bitter. This inquiry concluded that these changes are unlikely to reduce the acceptability of foods commonly offered to Indonesian infants and young children [16].

**Figure 1:**
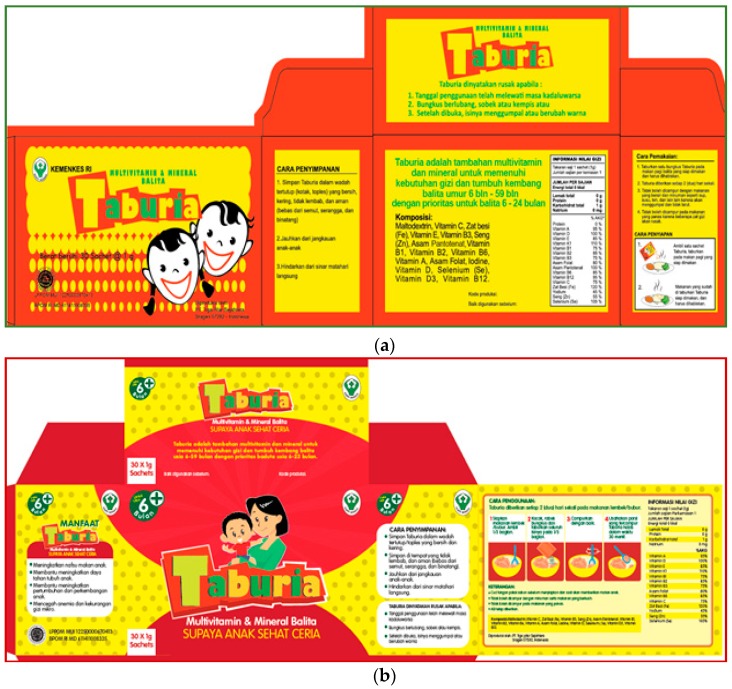
Packaging layout and messages (in Bahasa Indonesian) included in the box of *Taburia* containing 30 individual 1 g sachets. English translations of the messages are shown in the Supplementary Materials. (**a**) The original packaging approved by the Ministry of Health (MOH), decree number 2409 and enacted June 2011. (**b**) The improved packaging approved by the MOH, decree number 51 and enacted October 2016.

Recognizing the importance of clear information and education messages on the outer packaging of MNP products, which is often the most important material used in an MNP behaviour change intervention strategy [17,18], the Indonesian Ministry of Health (MOH) commissioned the Global Alliance for Improved Nutrition (GAIN) to improve the outer packaging of *Taburia*. Following this request, improvements of the type and form of information on the *Taburia* packaging and leaflet were conducted using the Home Fortification Technical Advisory Group (HF-TAG) [17] and GAIN (internal communication) guidelines on packaging development. Improvements to the *Taburia* outer packaging included more detailed instructions on the recommended use of *Taburia* (including target population and preparation instructions) and educational messages related to the reinforcement of adequate breastfeeding practices such as continued breastfeeding as recommended by the WHO and benefits of *Taburia* for the child. Changes were made to the content of the messages, as well as design (i.e., colour, shape, logo, illustration, pictograms, typography, and layout) based on packaging design principles used for commercial products [19,20]. The layout and messages of the original and improved *Taburia* packaging are shown in Figure 1. Caregiver acceptability of typography, colour schemes, pictograms and locally adapted phrases on a series of *Taburia* packaging proposals were evaluated during focus group discussions (FGD) to confirm appeal, attractiveness, preferences and clarity of the various components through 4 recorded FGD with >20 caregivers of children aged 6–24 months in the district of Sidoarjo in East Java. Caregivers provided feedback on specific components of the packaging, including spontaneous reactions, emotions and qualitative ratings. This process confirmed that the proposed modifications were in-line with cultural themes valued by community members.

In this context, we sought to investigate the influence of improved information and educational messages on the outer packaging of *Taburia* (as described in the Supplementary Materials) and cooking demonstrations on knowledge and adherence to recommended use among caregivers of children aged 6–36 months old one month after distribution. A mixed-methods approach including a community-based cluster randomized control trial followed by FGD was used. We hypothesized that the improved packaging would improve preparation practices and yield higher adherence to prescribed use.

## 2. Materials and Methods

### 2.1. Study Area and Population

The study was conducted from November 2015 to January 2016 in the sub-district of Tarik, in the district of Sidoarjo in East Java, Indonesia. Tarik has an estimated population of 70,000, many of whom are rice farmers [21]. Tarik has 20 villages with 73 active community health posts (*Posyandu*). Coverage of vitamin A distribution among children aged 6–59 months is >80% and more than two-thirds of children aged 0–23 months attend community health posts (*Posyandu*) for weight monitoring. Approximately half of the infants (55%) are exclusively breastfed for six months [21].

A total of 12 villages with similar social-demographic characteristics were purposely selected as study locations using the 2014 “Village Potential Statistics” (PODES) [22]. The variables used for the selection comprised the number of households, percentage of poor households, number of community health posts (*Posyandu*) per 1000 households and primary income of the majority of village residents. Potential study participants were identified from community health post (*Posyandu*) registries, with support from community health volunteers or health post personnel. All eligible caregivers with children aged 6–36 months (*n* = 1311) were invited to participate in the study. The following inclusion criteria were used to select children: (i) aged at least six months at baseline and <36 months at end-line; (ii) consuming foods other than breastmilk; (iii) not suffering from diarrhoea; (iv) not consuming medication prescribed by a medical doctor; (v) not receiving MNP in the last six months; and (vi) parental consent obtained. Children with low weight-for-age (*Z*-score < −2 with respect to the WHO Growth Standards) as reported in the community health post (*Posyandu*) charts were excluded because they are recipients of free fortified biscuits. To be eligible to participate, caregivers had to: (i) be aged 16–49 years old; (ii) be a permanent resident of the village; and (iii) have the capacity to understand the intent of the study to make an informed decision regarding consent.

### 2.2. Study Design

This community-based study included two main components: (i) a quantitative cluster randomized controlled trial (cRCT) to measure recommended use and intake adherence to prescribed *Taburia* use; and (ii) a qualitative component to explore factors affecting *Taburia* use among participants of each study group using FGD.

**Figure 2:**
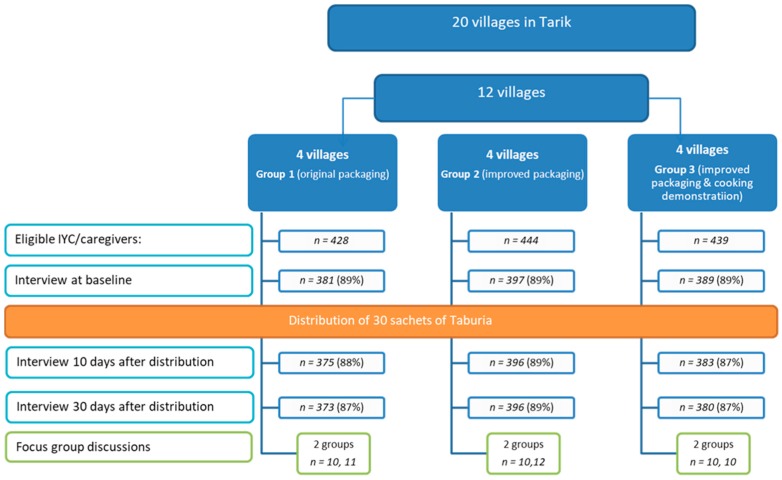
Study design and number of infants and young child/caregiver dyads participating in each study phase.

For the quantitative study, 1167 caregiver–child dyads were recruited at community health posts (*Posyandu*) (Figure 2). Caregiver–child dyads were randomized by their village (cluster) to receive 30 sachets of *Taburia:* (i) with the original outer packaging, as regulated by the MoH (Group 1); (ii) with the improved design and messages content of outer packaging (Group 2); or (iii) with the improved outer packaging combined with cooking demonstrations (Group 3). Cluster randomization by village was used to prevent caregivers from influencing each other.

For the follow-up qualitative study, a sub-sample of participants was purposely selected from each study group to investigate issues around perceptions of the packaging and use of *Taburia* by means of FGD.

Ethical clearance was obtained from the University Atmajaya, Jakarta (registration number 312/III/LPPM-PM.10.05/03/2015). Written permission to conduct fieldwork was obtained from East Java Provincial Government (10 November 2015), Sidoarjo District Government (12 November 2015) and Sidoarjo District Health Office (23 November 2015). All study participants signed informed consent forms.

### 2.3. Distribution of Taburia

Trained female field workers visited caregivers at their homes and distributed a single box of *Taburia* containing 30 individual 1 g sachets and an information leaflet during the first house visit. All field workers received four days of training and refresher sessions regarding study design, ethical responsibilities, mappings and listing of eligible participants, distribution of *Taburia* and interview and data-collection methodology. They were specifically instructed not to provide instructions on *Taburia* use, but to refer caregivers to the provided materials. Based on the recommended frequency of using *Taburia* every other day, the number of sachets needed to complement the diet of the index child over a period of 30 days is 15. However, caregivers were provided 30 sachets because this is the standard content of a *Taburia* box.

Caregivers were requested to follow the instructions provided on the outer packaging of the *Taburia* box and accompanying leaflet. The preparation instructions provided on outer box are shown in Table 1, and all additional product information and messages are shown in the Supplementary Materials. Improvements to the packaging included adding messages recommending *Taburia* use from six months onwards, using semi-solid foods as the food vehicle (rather than breakfast foods), mixing well, adding *Taburia* to 1/3 of the food portion prepared, consuming the preparation within 30 min and washing hands before preparing the food.

The content of *Taburia* sachets was identical for all groups. *Taburia* used in this study was formulated and developed by the central government of Indonesia. The premix for *Taburia* was supplied by DSM Nutritional Products Ltd. (Singapore) and packaged in Indonesia.

### 2.4. Cooking Demonstrations

Cooking demonstrations were held among Group 3 participants at the health post (*Posyandu*) in each village (*n* = 3). Participants were grouped according to the age of their child (6–8, 9–11, and 12–36 months) and shown how to prepare commonly consumed complementary foods with added *Taburia*. A staff member provided IYCF counselling, with an emphasis on adequate complementary feeding, and provided information about *Taburia* including proper use, expected benefits and possible side-effects. Additionally, participants were given a hand fan with key messages on *Taburia* benefits and use.

### 2.5. Data Collection

Field workers visited homes at baseline, 10 days and 30 days after distribution. Field workers used previously tested, structured data-collection tools to conduct face-to-face interviews with caregivers during each visit. The surveys were piloted in a population similar to the study population to ensure adequate comprehension.

At baseline, the survey included socio-demographic characteristics, exposure to supplementation programs (vitamin A and multivitamins in syrup, tablets or powders), exposure to IYCF messages, knowledge related to IYCF practices and iron deficiency and IYCF practices.

Ten days after receiving *Taburia*, caregivers were interviewed regarding use of *Taburia*. Field workers asked to see the box of *Taburia* and counted the number of used (empty) and unused (full) sachets and verified with caregivers whether used sachets were actually consumed by the index child.

Thirty days after exposure, use of *Taburia* was investigated as described above. In addition, caregivers were interviewed regarding knowledge of use of *Taburia*, sources of product information, preference for outer packaging (original compared to improved), food vehicles used for *Taburia*, perceived changes to the appearance and taste of foods with the addition of *Taburia* and side-effects.

Following individual interviews, FGD were performed for further in-depth exploration of experiences with *Taburia* use. Six FGD, two for each study arm, with 10–12 participants purposely selected among study respondents were undertaken. Topics explored included experiences with the preparation of foods with added *Taburia*, child acceptability of foods with added *Taburia*, sources of information used and preference of outer packaging (original vs. improved). FGD were conducted in the local language (Bahasa Indonesian) and were guided by a series of key themes. Discussions were voice recorded and all participants provided oral consent.

### 2.6. Data analysis and Outcome Measures

All data were checked for consistency during cleaning and analysis.

The primary outcome was adherence to prescribed use of *Taburia* during the one-month trial period. Although 30 sachets were distributed, adherence was defined as the proportion of the 15 sachets prescribed that were reportedly consumed by the target child. Adherence based on self-reported use and by observation was classified as “high” when 13–17 sachets were consumed by the targeted children (i.e., one sachet every other day, as prescribed), as “medium” when 8–12 or 18–22 sachets were consumed and as a “low” when ≤7 or ≥23 sachets were consumed.

FGD were recorded, transcribed and translated into English and coded through content analysis for concepts, dominant themes, and variability using NVivo qualitative data analysis Software; QSR International Pty Ltd. Version 11, 2015. Data were initially segmented by study group and then compared across groups.

### 2.7. Sample Size Calculation

Sample size estimates were based on the number of children needed in each group to detect (with a significance of *p* < 0.05 and power > 0.80) an effect size of >0.10 for reported adherence to prescribed use of *Taburia*, assuming an attrition rate of 15%. The sample size calculated was 356 caregiver–child dyads for each study group.

### 2.8. Statistical Analysis

Data analysis was performed by using IBM SPSS Statistic, Version 22.0. Baseline differences in socio-demographic characteristics and IYCF knowledge and practices between the three study groups were tested by using a Generalized Linear Model (GLM). A GLM was used to account for the effect of nested factors caused by the study design in which the 12 villages with a varying number of eligible caregiver–child dyads were randomly assigned into the three study arms. A nested design was used in the model because each village was assigned only to one treatment group. Depending on the distribution of the dependent variable, the following link functions were used: Binomial distribution via Logit function; Multinomial distribution via Cumulative Logit function; Normal distribution via Identity function and Gamma distribution via Log function. When significant differences were found between groups, post-hoc Wald’s Chi-square adjusted for multiple comparisons (least significant difference) were performed. Normality of residuals was tested using Kolmogorov–Smirnov and Shapiro–Wilks tests; Gamma distribution was used when non-normal residuals were observed.

To examine the differential effects of the intervention packages, main sources of information, preference of packaging and adherence to prescribed use of *Taburia* 10 and 30 days after distribution were examined using a nested design, GLM as described above. Odds ratios and 95% confidence intervals were calculated using a nested design in a Logistic Regression Model to compare adherence with prescribed use of *Taburia* 30 days after distribution among the three study groups.

## 3. Results

### 3.1. Response Rate

Of the 1311 caregiver–child dyads eligible for the study, 1167 (89%) were enrolled (Figure 2). Reasons for non-participation included refusal to participate (*n* = 70), caregiver was not found at home after three attempts (*n* = 29), caregiver was aged >49 years old (*n* = 18), child was aged >36 months at end-line (*n* = 18) or the child was underweight (*n* = 2). The number of caregiver–child dyads for the end-line survey (after 30 days) was 1149 (87% of those eligible). Loss to follow-up was due to the caregiver not being home after three attempts (*n* = 13 after 10 days and *n* = 5 after 30 days). Of the 380 caregivers invited to participate in the cooking demonstration, 298 (78%) attended the sessions.

### 3.2. Socio-Demographic Characteristics of Home-Trial Participants

The profiles of children and their caregivers were neither statistically (*p* > 0.05) nor substantially different between treatment groups (Table 2). The majority of caregivers (>90%) were the mother of the index child. Most caregivers were literate, with at least junior high school education and three-quarters were housewives.

### 3.3. Caregiver Knowledge Regarding Infant and Young Child Feeding Practices (Source: Baseline Interview)

Caregiver knowledge regarding IYCF practices and anaemia did not differ between treatment groups (*p* > 0.05) (Table 3). Although one-quarter of caregivers had heard messages related to adequate IYCF in last three months, only ~10% had received counselling on adequate IYCF practices from community health workers at health posts (*Posyandu*) in the previous three months. Approximately one-third could correctly define exclusive breastfeeding and <60% could report the recommended age for the introduction of complementary foods. Although almost 60% reported “meat, liver, or egg” and/or “green vegetables” as dietary sources of iron, only ~10% could report at least two symptoms of anaemia.

### 3.4. Infant and Young Child Feeding Practices (Source: Baseline Interview)

IYCF practices, as reported by participants, did not differ between treatment groups (*p* < 0.05) (Table 3). The majority of children included in the study were “ever” breastfed (>90%) and ~40% were breastfeeding at the time of the study. Approximately half the caregivers reported introducing semi-solids foods at six months of age as recommended by the WHO, but one-third of children were offered semi-solid foods prematurely and ~10% after six months. Two-thirds of children consumed family foods, and this proportion was significantly higher among children aged >12 months (*p* < 0.001). Few children consumed porridge the day before the interview. One-fourth of children were given iron supplements or multivitamin in the last six months.

### 3.5. Caregiver Knowledge Regarding Taburia Recommended Use (Source: Interview 30 Days after Distribution of Taburia + FGD)

**Figure 3:**
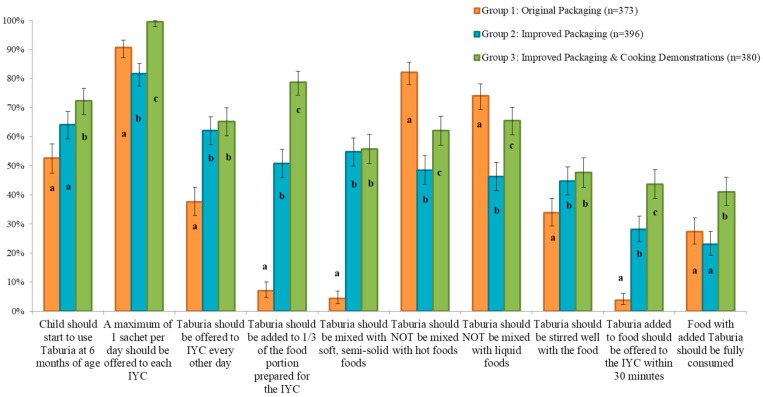
Caregiver knowledge regarding *Taburia* benefits and recommended use 30 days after distribution by study group. Group 1: Original outer packaging, as regulated by the MoH; Group 2: Improved design and messages content of outer packaging; Group 3: Improved outer packaging combined with cooking demonstrations. Error bars represent 95% confidence intervals. For all variables, differences between groups are statistically significant (*p* < 0.005) using a nested design, Generalized Linear Model (GLM) applying the Binomial distribution as base distribution via a Logit link function. Within a variable, values with different superscript letters are significantly different using Wald’s Chi-square adjusted for multiple comparisons (least significant difference) post-hoc analysis (*p*-value < 0.05).

The majority of caregivers in all groups (~90%) correctly reported that “a maximum of 1 sachet per day should be offered to each child” (Figure 3). Overall, knowledge was poorest regarding offering foods within 30 min from adding *Taburia*, and full consumption of foods with added *Taburia*.

Knowledge on use of *Taburia* according to the recommended instructions differed significantly between treatment groups (*p* < 0.05) and was generally better for the participants who received the improved outer packaging combined with cooking demonstrations (Group 3) (Figure 3). Caregivers who received the original packaging and no cooking demonstration (Group 1) scored less well than the other groups regarding knowledge that was not stated in the original packaging. Knowledge among Group 1 caregivers was particularly poor for the preparation instructions “*Taburia* should be added to 1/3 of the food portion prepared for the child”, “*Taburia* should be mixed with soft, semi-solid foods”, and “*Taburia* added to food should be offered to the child within 30 min”. Group 1 caregivers were also less knowledgeable on the instructions that “the child should start to use *Taburia* at 6 months of age”, “*Taburia* should be offered to child every other day”, and “*Taburia* should be stirred well with the food”. Although messages related to mixing *Taburia* with hot or liquid foods were explicitly included in both the original and improved packaging, knowledge that “*Taburia* should not be mixed with hot foods” and “*Taburia* should not be mixed with liquid foods” was better among caregivers who received the original packaging (Group 1). The original packaging offered reasons why these behaviours are recommended, as well as explicit examples of liquid foods to avoid mixing with *Taburia*, whereas the improved packaging only including the preparation recommendation.

During FGD, most caregivers in all study groups reported following the preparation instructions provided in the outer packaging and brochure because they had no previous experience with the product. Few caregivers reported using *Taburia* “based on their own experiences” with feeding young children. Caregivers generally felt confident that they were using *Taburia* as recommended, although some concerns were raised. A few caregivers reported being worried that the food vehicle was too hot, that *Taburia* made food stick to the spoon, or whether it could be given at the same time as milk. Caregivers were distressed by the recommendation that the food with added *Taburia* should be finished (as recommended in the original outer packaging), and even more distressed about the recommendation to “try to eat the *Taburia* mixed portion within 30 min”. Several caregivers appreciated the recommendation to mix *Taburia* with a proportion of the food offered to the child, but could not agree on whether it should be with half or one-third of food served. Further concerns regarded the safety of *Taburia*, such as whether it could be addictive, whether it was safe to give every day, if it caused more side-effects if offered at night and whether it would lead to obesity later in life.

### 3.6. Caregiver Experience with Use of Taburia (Source: FGD)

Focus group participants from all three home-trial study groups reported similar experiences with the use of *Taburia* during the previous month. Although the original *Taburia* packaging recommends the use of “ready-to-eat breakfast” and the improved packaging recommends the use of “semi-solid food/porridge” as food vehicle for *Taburia*, caregivers reported commonly adding *Taburia* to rice and other foods commonly consumed by their children. Rice, often mixed with other ingredients, was generally perceived as a convenient vehicle because it is readily consumed and liked by children. On the one hand, several caregivers reported that their child was too old to eat porridge and “mushy” foods. On the other hand, caregivers reported preparing porridge, or even buying commercial brands (Cerelac^®^) after reading the instructions solely to use it as a vehicle. *Taburia* was mostly offered to children for breakfast in the morning, as this is when children are hungriest. A single caregiver reported using half the *Taburia* sachet in the morning and the other half in the evening.

Several caregivers perceived changes in colour (such as brown spots) and taste (such as bitterness) of the foods with the addition of *Taburia*. A single caregiver reported that *Taburia* “smelled like medicine”. Some children initially rejected foods with added *Taburia*, but the sense was that they quickly got accustomed to it. In general, leftovers were disposed of.

Caregivers used several strategies to improve acceptability. The most commonly reported was the use of taste enhancers such as honey, soybean ketchup and commercial herbs, or offering palatable foods such as ice cream. A few caregivers reported varying the food vehicle used to avoid boredom, adding *Taburia* secretly or allowing the child to sprinkle the supplement her/himself. Some caregivers (from all study groups) reported adding *Taburia* to half or a third of their child’s food.

Caregivers reported that they perceived positive changes in their children with the use of *Taburia*. The most commonly reported were increased appetite, higher activity levels and improved immunity. Although side-effects such as diarrhoea and constipation were reported, caregivers reported that these issues did not persist and that their children needed an adaptation period to *Taburia*.

### 3.7. Main Source of Information on Use and Preparation of Taburia Used by Caregivers 30 Days after Distribution (Sources: Interview 30 Days after Distribution of Taburia and FGD)

Two-thirds of participants reported that they received general advice on IYCF verbally from health officials and trained cadres (Table 4). Fewer (~25%) relied on mass media communication channels or other means.

The main source of information for *Taburia* use was the outer packaging of *Taburia* (box holding 30 sachets), followed by the information brochure (Table 4). Group 1 caregivers relied mostly on the outer packaging (82%), and less on the information brochure (15%). Group 2 caregivers relied on the outer packaging (52%) and information brochure (40%) almost equally. Group 3 caregivers relied on both the outer packaging (40%) and information brochure (27%), but also on cooking demonstrations (11%), enumerator explanation (11%) and hand fan (8%).

During the FGD, caregivers reported that they relied on both the outer packaging of the *Taburia* box and the brochure for instruction on how to use *Taburia*, but much less on the individual sachet or fan. The brochure was appreciated because it was considered more comprehensive, but caregivers were concerned that not all caregivers would receive one or that it could be easily misplaced. The individual sachet was not used because “it lacked information” and the “font size is too small”, and the fan, used as a children’s toy, was easily misplaced. Caregivers who attended the cooking demonstration (Group 3) considered the practical information provided very useful, and appreciated the examples of food vehicles that can be used.

Although the information materials were appreciated, most caregivers considered it critical to receive instructions on the use of *Taburia* from an “expert”, a person they could trust. This was considered essential to “build trust in *Taburia*” and for caregivers who could not read. Further concerns included the distribution of “fake products” and the numerous brands of supplements available in local pharmacies which could cause confusion.

### 3.8. Preference of Outer Packaging of Taburia 30 Days after Distribution (Sources: Interview 30 Days after Distribution of Taburia and FGD)

As reported in Table 4, the vast majority of participants in all treatment groups (85%) preferred the improved package message design and content over the original package (*p* < 0.001). Reported reasons included the attractive colour of the packaging (67%), better information on use and preparation of *Taburia* (56%) and better information on benefits of *Taburia* (21%).

During the FGD, there was a unanimous preference for the improved outer packaging of *Taburia*. Caregivers preferred the design as it was considered “more colourful”, “more attractive”, “cleaner” and “more readable due to the larger font size”. Caregivers also preferred the images used in the new packaging because the woman depicted looked “nicer” and “happier”. The diagrams about instruction for use were considered essential given that “some caregivers cannot read”. Furthermore, caregivers preferred the messages on the improved packaging, as this was considered “more complete”. Valued information included the age of the children that benefit from the use of *Taburia*, the suggestion to add *Taburia* to a third of the food prepared and a list of benefits to the child.

### 3.9. Reasons for Offering Taburia (Sources: Interview 30 Days after Distribution of Taburia and FGD)

During the 30-day study, reported reasons for offering *Taburia* were related to benefits observed in the children. These included increased appetite (76%), stronger, healthier and happier children (36%), improved immunity (32%), prevention of anaemia (17%), increased growth (16%) and cognitive development (36%). These benefits were explicitly stated in the improved outer packaging, but also in the original and improved brochure.

FGD highlighted caregivers’ willingness to offer *Taburia* to their child because it had been recommended by the study enumerator. The enumerators were considered important to gain trust in the product and address concerns about cost and quality.

### 3.10. Adherence to Prescribed Use of Taburia 10 and 30 Days after Distribution (Sources: Interview 30 Days after Distribution of Taburia and Counting of Sachets)

The majority of participants (>97%) reported offering *Taburia* to the index child 10 and 30 days after receiving the supplement (Table 5). Sharing of *Taburia* with family members or neighbours was reported by ~10% of caregivers. At the end of the 30-day trial, >75% of caregivers in all groups reported that the last meal offered to the child contained *Taburia*.

Ten days after distribution, *Taburia* consumption reportedly occurred every other day (as recommended) among 40% of Group 1, 52% of Group 2 and 66% of Group 3 caregivers. Furthermore, “underconsumption” of *Taburia* (seven or fewer sachets) was much less prevalent than “overconsumption” (23 sachets or more). “Overconsumption” was especially high among Group 1 participants (33% in Group 1 versus 15% in Group 2) (Table 5).

**Figure 4:**
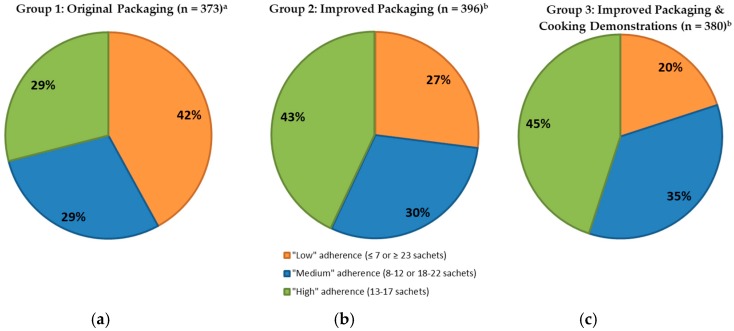
Adherence to prescribed use of Taburia 30 days after distribution among children who consumed *Taburia* without sharing. Adherence was defined as “high” (13–17 sachets), “medium” (8–12 or 18–22 sachets) or “low” (≤7 or ≥23 sachets). (**a**) Group 1: Original outer packaging, as regulated by the MoH; (**b**) Group 2: Improved design and messages content of outer packaging; and (**c**) Group 3: Improved outer packaging combined with cooking demonstrations. Differences between groups are statistically different (*p* < 0.001) using multinomial distribution via Cumulative Logit function. Values with different superscript letters are significantly different using Wald’s Chi-square adjusted for multiple comparisons (least significant difference) post-hoc analysis (*p*-value < 0.05).

Thirty days after distribution, adherence was lower than after 10 days (*p* < 0.001) and 29% of Group 1, 43% of Group 2 and 45% of Group 3 caregivers reported offering 13–17 sachets of *Taburia* (defined as “high” adherence) to the index child (Figure 4). Adherence to prescribed use was lower among Group 1 study participants when compared to Groups 2 and 3 (*p* < 0.001 after 10 days, *p* = 0.005 after 30 days), whereas no differences were observed between Groups 2 and 3 (*p* = 0.545 after 10 days, *p* = 0.523 after 30 days).

**Figure 5:**
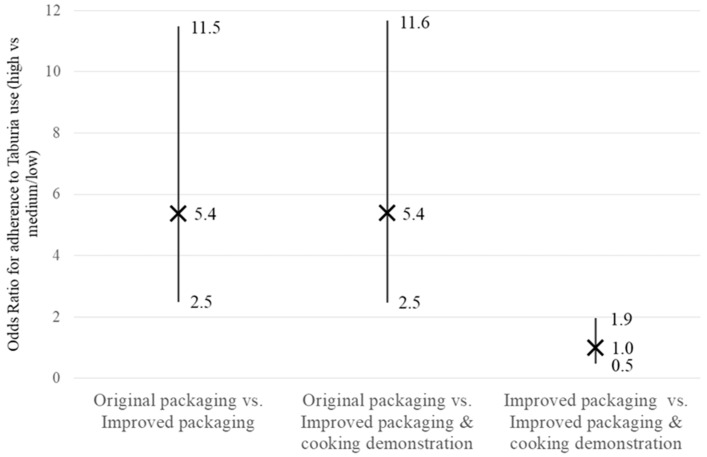
Odds ratios comparing “high” (13–17 sachets) versus “medium/low” (≤12 or ≥18 sachets) adherence with prescribed use of *Taburia* 30 days after distribution among the three study groups. Group 1: Original outer packaging, as regulated by the MoH; Group 2: Improved design and messages content of outer packaging; and Group 3: Improved outer packaging combined with cooking demonstrations. Odds ratio pairwise comparisons were performed using a nested design in a Logistic Regression Model. The X represents the odds ratio and the error bars represent 95% confidence intervals.

As shown in Figure 5, caregivers who received the improved packaging (with or without a cooking demonstration) were five times more likely [odds ratio 5.4 (2.5–11.5)] to have “high” adherence to prescribed use of *Taburia* (defined as 13–17 sachets versus fewer or more 30 days after distribution) when compared to those who received the original packaging. However, having participated in cooking demonstrations did not increase the likelihood of “high” adherence among caregivers who received the improved packaging [odds ratio 1.0 (0.5 to 1.9)].

## 4. Discussion

Several studies describe the development and pre-testing of MNP packaging, as well as program experiences with specific aspects of packaging and how these affect product perceptions, illustrating the importance of adequate, culturally appropriate packaging [23,24,25]. To our knowledge, this is the first study specifically designed to quantify the effect of improved design, information and educational messages of MNP outer packaging on knowledge and recommended use of MNP in a programmatic context. We demonstrated that the content and design of MNP packaging can have a significant influence on knowledge of, and adherence to, its recommended use.

The outer packaging of *Taburia* distributed in this study recommended offering *Taburia* to the child “once every 2 days”. Interestingly, several caregivers offered *Taburia* almost daily; especially among Group 1 study participants (14% offered 28 sachets or more the previous month). Possible explanations for daily use of *Taburia* include the distribution of a 30-sachet *Taburia* box which is meant to last two months, previous MOH recommendation for daily use of MNP, the perception that “more is better” and the conceptually easier regimen of a daily routine. Differences between groups may be explained by the greater clarity of the messages in the improved packaging, greater reliance of Group 2 participants on the supplementary brochure which includes additional information and reinforcement of recommended *Taburia* use during cooking demonstrations in Group 3. Consequently, “low” adherence (defined as having offered ≤7 or ≥23 sachets in the previous month) consisted predominantly of “overconsumption” of *Taburia*, especially among Group 1 participants. It is important to note that, whereas low consumption of *Taburia* will attenuate the expected outcome of MNP supplementation, daily consumption is expected to be beneficial to the child’s health. Nevertheless, in our study, consumption frequency above the recommendation was classified as “low” adherence because we examining adherence to instructions provided.

### 4.1. Publications Describing the Importance of Culturally Sensitive MNP Packaging

Various studies confirm the critical importance of culturally appropriate packaging that carries clear and self-explanatory messages informing beneficiaries of the content, target group, preparation procedures, and frequency of use of the product to achieve higher MNP acceptance in a programmatic context [23,24,25,26,27,28,29]. These studies describe the importance of considering local context, cultural practices, and beliefs in developing the packaging. Packaging aspects such as colour scheme, type of picture or image used, and wording of messages have different associations and differing interpretations across cultures, influencing adoption and appropriate use of MNP.

Examples of specific package MNP characteristics that influence caregivers in Laos include the use of colours that reflect patriotism and an instructional image showing a caregiver actively feeding her healthy child [30]. Packaging attributes associated with a healthy and a clever child were well received and a series of images showing healthy development from six months onwards conveyed the benefits of continued use. Lastly, caregivers valued pictorial instructions for use over written instructions. Examples of packaging influences in Kenya include association of the colour red with improved blood (i.e., less anaemia) and a misunderstanding about food preparation due to the use of a picture of a bowl which was identified as a family pot rather than a child’s serving vessel [24]. Formative research to inform the design of an MNP program in Timor-Leste highlights the feasibility and importance of involving caregivers in choosing a locally appropriate name, packaging design, content and promotional messages for MNP in-home fortification programs [25]. The authors suggest that the MNP product should be given a local name associated with improved growth and health, that packaging be orange in colour with a picture of a “big” child and a locally known complementary food associated with healthy children.

MNP packages are not always well received, especially in populations with low literacy [28] and culturally insensitive aspects of packaging can lead to misconceptions and poor use. In Kenya, for example, lack of culturally sensitive packaging design led to misinformation regarding its intended purpose. Some beneficiaries believed that the product might be a contraceptive, others that it was a medicine and some suspected that that the contents might have been derived from deleterious ingredients [23]. The package depicted a family with a single child in a setting where large families are the norm, and the individual sachet resembled a condom packaging.

Although MNP packaging is hugely important, several studies stress the importance of adequate accompanying communications materials, a social marketing campaign, and training for those who will provide the product [26]. A review of factors affecting MNP acceptability stresses the prominent role of interpersonal communication by frontline workers whose activities may include cooking demonstrations, home visits, and counselling [18]. In our study, although the improved packaging information was considered comprehensive, easy-to-follow and user-friendly, caregivers still preferred to receive the product from a trusted source along with reassurance of the product’s safety and benefits to the child.

### 4.2. Importance of MNP Packaging as Communication Tool

In the commercial sector, the role of packaging on consumer decisions is a crucial marketing and communications tool. It is widely recognized that packaging differentiates a product from others, creates brand recognition which improves trustworthiness and influences the decision of the consumers at the point-of-sale as well as at the point-of-use [31]. It is estimated that approximately 70 percent of all purchase decisions on goods are made at point-of-purchase [32]. In marketing, the need to consider beauty and art in the design of the outward features of the product is indispensable, but not all aesthetic package design elements trigger consumer purchase behaviour, thus requiring careful thought to specific aspects [33]. Food packaging design has become an integral part of marketing and is paramount to the promotion and communication around a specific product. Improved packaging quality can potentially attract more consumers towards the product, improve in-home use experience, and increase the chances of repurchase.

MNP are no exception to the rules that are applicable to the packaged goods industry and are critical for both commercially available (for sale) and MNP distributed for free. MNP packaging information provides a communication platform to share information directly with caregivers at the critical point of use. MNP packaging is often the first and always the last point of contact, and often may be the only information the caregiver receives. Although the individual sachet provides the most secure point of contact, it has limited space to capture information and the content is often restricted by the distributors. Outer packaging, usually designed to hold a monthly supply of sachets, can accommodate more information. It is therefore important that both sachet and outer packaging contain the critical information and steps for appropriate use, and that the messages remain consistent. MNP packaging material is especially relevant in situations in which key aspects of program implementation are weak, for example, in contexts where community health workers receive limited training [23]. Our study confirms that the outer packaging is the main source of information used for instructions on MNP use (for 82% of caregivers who received the original packaging and 52% of caregivers who received the improved packaging), but that the accompanying information brochure was also consulted (by 15% of caregivers who received the original brochure and 40% of caregivers who received the improved brochure).

### 4.3. Guidelines for the Development of Effective MNP Packaging

MNP packaging information must be complete, accurate and easy-to-understand within its context of use. Developing locally appropriate MNP packaging is a lengthy process that requires formative research, multiple rounds of revisions and testing in a field setting. At the same time, key features of MNP packaging should always be present regardless of the context. These include a locally relevant and appropriate product name, clear messaging that the product is not for use by infants under six months of age and a statement supporting the importance of continued breastfeeding to two years of age and beyond. Although there are no official guidelines regarding the information to be featured on the packaging, HF-TAG provides a series of practical recommendations giving guidance on key features of packaging, and how to ensure that the product is not used incorrectly, such as given with a bottle, and that no false health claims are made [17]. They also include pictograms that illustrate comprehensively how to use the product, and that are easily understood by illiterate caregivers across multiple countries.

### 4.4. Study Limitations

This study design had some limitations: (i) 12 or 20 villages in the sub-district of Tarik were purposely selected as study locations, thus the sample is not representative of the area; (ii) the study was not double-blinded, both the researchers and the participants were aware of intervention assignment, and no placebo was used; (iii) although triangulated with observation, adherence to prescribed use of *Taburia* was based on self-reports; and (iv) the duration of the trial was 30 days and the long-term effect of the improved packaging remains uncertain.

## 5. Conclusions

Our findings demonstrate that outer MNP packaging design and comprehensiveness of information provided are important influencers of recommended use of MNP. This study provided the evidence requested by the Indonesian MOH to justify a new national standard for MNP packaging for distribution in national programs. The importance of MNP packaging is likely to apply to beyond the sub-district of Tarik and Indonesia.

## Figures and Tables

**Table 1:** Directions for use of *Taburia* in the original and improved Taburia packaging information on outer box.

Instruction	Original *Taburia*	Improved *Taburia*
Recommended age to start offering *Taburia*	-	“*For age +6 months*”
Prescribed frequency of use of *Taburia*	*“Give Taburia once every 2 days”*	*“Give Taburia once every 2 days”*
Images included on packaging	Two pictograms illustrate preparation.	Four elaborate pictograms illustrate preparation.
	Image 1 shows Taburia being sprinkled on a meal on a plate. Image 2 shows the meal on a plate.	**Image 1** shows a bowl in which 1/3 of the meal is set aside. **Image 2** shows *Taburia* being sprinkled onto 1/3 of the meal. **Image 3** shows a spoon stirring *Taburia* with 1/3 of the meal. **Image 4** shows a spoon being used to feed the meal.
Preparation instructions	*“Sprinkle one sachet of Taburia…”*	*“Shake, tear the sachet and sprinkle all the content...”*
	*“… on prepared breakfast”*	*“Prepare semi-solid food/porridge”*
		*“ … to the 1/3 portion”*
		*“Mix well”*
	*“Ensure that the food with added Taburia is fully consumed”*	*“Ensure that the food with added Taburia is consumed within 30 min”*
	*“Do not mix with liquid foods and drinks such as soup, milk, or tea to prevent clotting”*	*“Do not mix with liquid foods and drinks”*
	*“Do not mix with hot foods because some nutrients will be destroyed”*	*“Do not mix with hot foods”*

**Table 2:** Baseline socio-demographic characteristics of study participants.

	Group 1		Group 2		Group 3		*p*-Value ^4^
	(Original Packaging) ^1^		(Improved Packaging) ^2^		(Improved Packaging and Cooking Demonstrations) ^3^		
	(*n* = 373)		(*n* = 396)		(*n* = 380)		
	*n*	%	*n*	%	*n*	%	
Child characteristics:							
Sex							
Boy	200	54	206	52	186	49	0.826 ^BL^
Girl	173	46	190	48	194	51	
Age group							
6–11 months	80	21	81	20	84	22	0.424 ^MC^
12–23 months	147	39	159	40	162	43	
24–36 months	146	39	156	39	134	35	
Caregiver characteristics:							
Caregiver							
Mother	345	92	365	92	352	93	0.320 ^MC^
Grandparent	14	4	12	3	9	2	
Other	14	4	19	5	19	5	
Age in years							
16–19 (teenagers)	3	1	8	2	3	1	0.242 ^GL^
20–24 y	89	24	92	23	91	24	
25–29 y	83	22	94	24	112	29	
30–34 y	124	33	115	29	102	27	
35–39 y	39	10	49	12	36	9	
40–49 y	35	9	38	10	36	9	
Formal schooling completed by the mother							
Elementary school or lower	28	8	36	9	33	9	0.141 ^MC^
Junior high school or equivalent	115	31	125	32	134	35	
Senior high school or higher	230	62	235	59	213	56	
Mother’s occupation							
Housewife	286	77	304	77	272	72	0.838 ^MC^
Works full-time	57	15	59	15	59	16	
Works part-time	30	8	33	8	49	13	
Possession of goods ^5^							
Quartile 1 (<26)	197	53	199	50	176	46	0.124 ^MC^
Quartile 1 (26–50)	20	5	19	5	16	4	
Quartile 1 (51–75)	124	33	138	35	157	41	
Quartile 1 (76–100)	32	9	40	10	31	8	

^1^ Original outer packaging, as regulated by the Indonesian MoH; ^2^ Improved design and messages content of outer packaging; ^3^ Improved outer packaging combined with cooking demonstrations; ^4^ Using a nested design, Generalized Linear Model (GLM) applying the following base distribution link functions: ^BL^ Binomial distribution via Logit function; ^MC^ Multinomial distribution via Cumulative Logit function; and ^GL^ Gamma distribution via Log function; ^5^ Based on ownership of 12 key assets.

**Table 3:** Infant and young child feeding knowledge and practices at baseline.

	Group 1		Group 2		Group 3		*p*-Value ^4^
	(Original Packaging) ^1^		(Improved Packaging) ^2^		(Improved Packaging and Cooking Demonstrations) ^3^		
	(*n* = 373)		(*n* = 396)		(*n* = 380)		
	*n*	%	*n*	%	*N*	%	
Caregiver knowledge							
Heard message related to adequate IYCF in last 3 months	101	27	91	23	91	24	0.206 ^BL^
Was counselled about adequate IYCF in last 3 months	35	9	56	14	44	12	0.078 ^BL^
Correctly reported the meaning of “exclusive breastfeeding”	137	37	117	30	105	28	0.123 ^BL^
Correctly reported the age at which complementary foods should be introduced (at six months)	220	59 ^a^	244	62 ^a^	201	53 ^b^	0.026 ^BL^
Reported “meat, liver, egg” and/or “green vegetables” as dietary sources of iron	219	59	225	57	195	51	0.273 ^BL^
Reported at least 2 symptoms of anaemia	54	14	42	11	44	12	0.092 ^BL^
Infant and young child feeding practices							
Ever breastfed	345	92	357	90	349	92	0.409 ^BL^
Currently being breastfed	155	42	155	39	156	41	0.932 ^BL^
Age at which semi-solid foods were introduced:							
Before six months of age	97	26	125	32	132	35	0.060 ^MC^
At six months of age	210	56	208	53	192	51	
After six months of age	65	17	63	16	56	15	
Consumes family foods (i.e., foods are not processed for the child)	242	65	266	67	243	64	0.815 ^BL^
Was fed home-made porridge yesterday	26	7	30	8	29	8	0.851 ^BL^
Was fed commercial porridge yesterday	57	15	55	14	56	15	0.852 ^BL^

^1^ Original outer packaging, as regulated by the Indonesian MoH; ^2^ Improved design and messages content of outer packaging; ^3^ Improved outer packaging combined with cooking demonstrations; ^4^ Using a nested design, Generalized Linear Model (GLM) applying the following base distribution link functions: ^BL^ Binomial distribution via Logit function and ^MC^ Multinomial distribution via Cumulative Logit function. Within a row, values with different superscript letters are significantly different using Wald’s Chi-square adjusted for multiple comparisons (least significant difference) post-hoc analysis (*p*-value < 0.05).

**Table 4:** Main source of information on use and preparation of *Taburia* used by caregivers and preference of packaging 30 days after distribution.

	Group 1		Group 2		Group 3		*p*-Value ^4^
	(Original Packaging) ^1^		(Improved Packaging) ^2^		(Improved Packaging and Cooking Demonstrations) ^3^		
	(*n* = 373)		(*n* = 396)		(*n* = 380)		
	*n*	%	*n*	%	*n*	%	
Sources of information used for infant and young child health advice							
Verbal (health official and trained cadres)	247	66 ^a^	281	71 ^b^	221	58^ a^	0.002 ^BL^
Television, Radio	107	29^ a^	125	32^ a^	56	15 ^b^	<0.001 ^BL^
Community meeting	85	23^ a^	77	19^ a^	114	30 ^b^	<0.001 ^BL^
Printed media (brochure, poster, etc.)	116	31^ a^	67	17 ^b^	78	21 ^b^	<0.001 ^BL^
Internet	94	25^ a^	91	23^ a,b^	65	17 ^b,c^	0.048 ^BL^
Demonstrations	16	4^ a^	14	4 ^b^	83	22 ^c^	<0.001 ^BL^
Visual Media	40	11	15	4	21	6	0.218 ^BL^
Public figure/religious person/elderly	27	7	23	6	39	10	0.074 ^BL^
Main source of information used for instructions of *Taburia* use							
Outer packaging of *Taburia* (box holding 30 sachets)	306	82	207	52	153	40	<0.001 ^MC^
*Taburia* information brochure	56	15	157	40	102	27	
Individual *Taburia* sachet (1 g sachet)	8	2	5	1	11	3	
Enumerator explanation	3	1	3	1	40	11	
Hand fan	NA	-	24	6	30	8	
Cooking demonstration	NA	-	NA	-	43	11	
(*post-hoc analysis*)		^a^		^b^		^c^	
Preference of outer packaging of *Taburia* (box holding 30 sachets)							
Original package message content	44	12	75	19	56	15	0.346 ^BL^
Improved package message content	329	88	321	81	324	85	
Reasons reported for preference:							
More attractive colour	279	75^ a^	259	65 ^b^	234	62 ^b^	0.001 ^BL^
Better information on use and preparation of *Taburia*	239	64^ a^	174	44 ^b^	234	62^ a^	<0.001 ^BL^
Better information on benefits of *Taburia*	94	25^ a,c^	77	19 ^b,c^	76	20 ^c^	0.014 ^BL^
Picture showing child with mother	50	13^ a^	110	28 ^b^	85	22 ^b^	<0.001 ^BL^

^1^ Original outer packaging, as regulated by the MoH; ^2^ Improved design and messages content of outer packaging; ^3^ Improved outer packaging combined with cooking demonstrations; ^4^ Using a nested design, Generalized Linear Model (GLM) applying the following base distribution link functions: ^BL^ Binomial distribution via Logit function and ^MC^ Multinomial distribution via Cumulative Logit function. Within a row, values with different superscript letters are significantly different using Wald’s Chi-square adjusted for multiple comparisons (least significant difference) post-hoc analysis (*p*-value < 0.05).

**Table 5:** Adherence with prescribed use of Taburia 10 and 30 days after distribution.

	Group 1		Group 2		Group 3		*p*-Value ^4^
	(Original Packaging) ^1^		(Improved Packaging) ^2^		(Improved Packaging and Cooking Demonstrations) ^3^		
	(*n* = 373)		(*n* = 396)		(*n* = 380)		
	*n*	%	*n*	%	*n*	%	
10 days after distribution							
*Taburia* was offered to the index child in the last 10 days	367	98	388	98	378	99	1.000 ^BL^
Caregiver reported sharing *Taburia* with other family members of neighbours	23	6	24	6	31	8	0.240 ^BL^
Number of sachets used in the last 10 days among children who consumed *Taburia* without sharing							
None	5	1	8	2	2	1	<0.001 ^GL^
1 to 3 sachets	96	27	111	30	77	22	
4 to 6 sachets	141	40	193	52	231	66	
7 to 9 sachets	50	14	25	7	24	7	
10 or more sachets	58	17	35	9	15	4	
(*post-hoc analysis*)		^a^		^b^		^b^	
30 days after distribution							
*Taburia* was offered to the index child in the last 20 days	359	96	384	97	371	98	1.000 ^BL^
Caregiver reported sharing *Taburia* with other family members of neighbours	40	11	36	9	52	14	0.082 ^BL^
Number of sachets used in the last 30 days among children who consumed *Taburia* without sharing							
Less than 3 sachets	11	3	10	3	6	2	0.007 ^MC^
3 to 7 sachets	21	6	30	8	19	6	
8 to 12 sachets	51	15	38	11	57	17	
13 to 17 sachets	96	29	155	43	148	45	
18 to 22 sachets	45	14	71	20	57	17	
23 to 27 sachets	62	19	41	11	33	10	
More than 27 sachets	47	14	15	4	8	2	
(*post-hoc analysis*)		^a^		^b^		^b^	

^1^ Original outer packaging, as regulated by the MoH; ^2^ Improved design and messages content of outer packaging; ^3^ Improved outer packaging combined with cooking demonstrations; ^4^ Using a nested design, Generalized Linear Model (GLM) applying the following base distribution link functions: ^BL^ Binomial distribution via Logit function; ^MC^ Multinomial distribution via Cumulative Logit function; ^NI^ Normal distribution via Identity function and ^GL^ Gamma distribution via Log function. Within a row, values with different superscript letters are significantly different using Wald’s Chi-square adjusted for multiple comparisons (least significant difference) post-hoc analysis (*p*-value < 0.05).

## Supplementary Materials

The following are available online at http://www.mdpi.com/2072-6643/10/6/747/s1, Table S1: Original and improved *Taburia* packaging information on outer box and brochure, and the HF-TAG and GAIN guidelines for MNP packaging.
