# Supplementary material for: Improved Information and Educational Messages on Outer Packaging of Micronutrient Powders Distributed in Indonesia Increase Caregiver Knowledge and Adherence to Recommended Use
Source: Nutrients. 2018 Jun 8;10(6):747. doi: 10.3390/nu10060747 (PMC6024872; doi:10.3390/nu10060747)
Supplement: Supplementary file 1 [file nutrients-10-00747-s001.pdf]

Supplementary Materials: Table S1: Original and improved Taburia packaging information on outer box and brochure, and the HF-TAG and GAIN guidelines for MNP packaging.

	HF-TAG Guidelines [1]	GAIN Guidelines [2]	*"Taburia"* Packaging Information on Outer Box (Which Holds 30 Sachets)		*"Taburia"* Packaging Information on Sachet (1 g Single Dose)	*"Taburia"* Information on Brochure	
			**Original Taburia Package [3]**	**Improved Taburia Package [4]**		**Given with Original Taburia**	**Given with Improved Taburia**
**Product name**	A locally relevant and appropriate product name	Simple yet meaningful in local context, e.g., "Bibo" is commonly one of baby's first words in Vietnamese	*"Taburia"*	*"Taburia"*	*"Taburia"*	*"Taburia"*	*"Taburia"*
**Product description/Target age**	Clear messaging that the product is not for use by children under six months of age	Clearly specify target group "for 6 months and over", written and/or through illustration	-	*"For age +6 month"*. This is stated on all sides of the box	-	-	*"For age +6 month"*. This is stated on all sides of the box
	A text specifying the age range of the intended user		*"Taburia is supplemental multivitamins and minerals to fulfil the nutritional and developmental requirement of children aged 6–59 months, with priority on babies aged 6–23 months"*	*"Taburia is supplemental multivitamins and minerals to fulfil the nutritional and developmental requirement of children aged 6–59 months, with priority on babies aged 6–23 months"*	*"Taburia is supplemental multivitamins and minerals to fulfil the nutritional and developmental requirement of children aged 6–59 months, with priority on babies aged 6–23 months"*	*"Taburia is supplemental multivitamins and minerals to fulfil the nutritional and developmental requirement of children aged 6–59 months, with priority on babies aged 6–23 months"*	*"Taburia is supplemental multivitamins and minerals to fulfil the nutritional and developmental requirement of children aged 6–59 months, with priority on babies aged 6–23 months"*
**Continued breastfeeding**	A statement supporting the importance of continued breastfeeding to		-	*"Continue to breastfeed"*	-	-	*"Continue to breastfeed"*

	two years of age and beyond						
**Slogan**	A strategic slogan for promotion	Use slogan clearly stating what the product is for and its merit, e.g., "Providing vitamins and minerals"	*"Children U5 Multivitamin and Mineral"*	*"Children U5 Multivitamin and Mineral"*	-	*"Children U5 Multivitamin and Mineral"*	*"Children U5 Multivitamin and Mineral"*
			-	*"For a Healthy Cheerful Child"*	-	*"For a Healthy Cheerful Child"*	*"For a Healthy Cheerful Child"*
**Health claims/Benefits of** *Taburia*	Do not state the following:<br>- MNP improve growth<br>- MNP give energy<br>- MNP make children smarter/more clever.<br>It might be permissible to mention that vitamins and minerals are important contributors to children's healthy growth and development, including cognitive development, but that vitamins and minerals do not contain or give energy (calories).	*"Prevents anaemia"*<br>*"Supports healthy development"*<br>*"Supports brain development"*<br>*"Improves child's appetite"*<br>*"Improves nutrient intake"*<br>Beware of unverifiable claims such as "more energy and growth"	*"… to fulfil the nutritional and developmental requirement …"*	*"…to fulfil the nutritional and developmental requirement …"*	*"…to fulfil the nutritional and developmental requirement …."*	*"… to fulfil the nutritional and developmental requirement …"*	*"… to fulfil the nutritional and developmental requirement …"*
			-	*"Improves child appetite"*	-	*"Improves child appetite"*	*"Improves child appetite"*
			-	*"Helps to improve child's immune system"*	-	*"Child is sick less easily"*	*"Helps to improve child's immune system"*
			-	*"Helps to improve child's growth and development"*	-	*"Child's Growth and Development will be in accordance to the child's age"*	*"Helps to improve child's growth and development"*
			-	*"Prevents anaemia and micronutrient deficiencies"*	-	*"Child is not anaemic so they are more intelligent and cheerful"*	*"Prevents anaemia and micronutrient deficiencies"*
			-	-	-	*"Taburia is one way to overcome anaemia in U5 children"*	*"Taburia is one way to overcome anaemia in U5 children"*
			-	-	-	*"Foods consumed by children U5 tend to be poor in iron and other nutrient. Taburia helps children U5 obtain important micronutrients (vitamins and mineral)"*	*"Foods consumed by children U5 tend to be poor in iron and other nutrient. Taburia helps children U5 obtain important micronutrients (vitamins and mineral) "*
			-	-	-	*"Contains 12 essential vitamins and 4 essential minerals"*	*"Contains 12 essential vitamins and 4 essential minerals"*
**Product claims**			-	-	-	"Does not change children's eating habits"	"Does not change children's eating habits"
			-	-	-	*"Practical"*	*"Practical"*

		-	-	-	*Halal, does not contain traces of alcohol and pork.*	*Halal, does not contain traces of alcohol and pork.*
**Symptoms of anaemia in infants and young children**		-	-	-	*"Inhibits the development of children u5"*	*"Inhibits the development of children u5"*
		-	-	-	*"Inhibits the motoric development of children u5 (sluggish, not cheerful)"*	*"Inhibits the motoric development of children u5 (sluggish, not cheerful)"*
		-	-	-	*"Reduces intelligence of children u5"*	*"Reduces intelligence of children u5"*
		-	-	-	*"Decrease the immune system so children u5 are easily sick"*	*"Decrease the immune system so children u5 are easily sick"*
**Side-effects**		-	-	-	"Don't worry …"	"Don't worry …"
					"… Taburia is not addictive"	*"… Taburia is not addictive"*
		-	-	-	*"… Taburia can be consumed on a regular basis as instructed"*	*"… Taburia can be consumed on a regular basis as instructed"*
		-	-	-	*"… if the child's stool become dark in colour"*	*"… if the child's stool become dark in colour"*
		-	-	-	*"… If the child is constipated, offer a lot of boiled water"*	*"… If the child is constipated, offer a lot of boiled water"*
		-	-	-	*"… if the child has diarrhoea, take the child to the health worker"*	*"… if the child has diarrhoea, take the child to the health worker"*
		-	-	-	*"… if the colour/taste/flavour of the food changes slightly, do not worry as the changes will not decrease the benefits of Taburia"*	*"… if the colour/taste/flavour of the food changes slightly, do not worry as the changes will not decrease the benefits of Taburia"*
**Language**	Text printed in the language best known to the beneficiaries	All information is provided in the local language	All information is provided in the local language	All information is provided in the local language	All information is provided in the local language	All information is provided in the local language
**Images**	If images of children are used, they should be older than six months.	An illustration of two children older than six months is shown (the girls has braids in her hair)	An illustration a mother and a child older than six months is shown (child is sitting up well and has teeth)	An illustration of two children older than six months is shown (the girls has braids in her hair)	An illustration a mother and a child older than six months is shown (child is sitting up well and has teeth)	An illustration a mother and a child older than six months is shown (child is sitting up well and has teeth)

	When using images on the box or sachet, children should be avoided. If an image of a child is used, it should be free standing (not being held or sitting). This ensures that there is no ambiguity around the child's age, as no child under six months of age would be able to stand on its own. Another strategy is to depict children with teeth, as few children have teeth before six months of age.		Caricature of a girl and boy, showing only their faces. The girl has braids and both look older than 6 months	Caricature of a mother holding her child. The child looks older than 6 months because he is sitting up firmly	No illustration	Caricature of a mother holding her child. The child looks older than 6 months because he is sitting up firmly. Caricature of boy alone, in one he looks sad, in the other he is full of energy. Children are standing alone and have a lot of hair, the girl has pigtails. There are also pictures of the Taburia box and sachets. There are images of pests (rat and coach roach) eaten Taburia	Caricature of a mother holding her child. The child looks older than 6 months because he is sitting up firmly. Caricature of mother alone, boy alone and girl alone. Children are standing alone and have a lot of hair, the girl has pigtails. There is also a picture of the Taburia box
**Appearance/layout**	Background design could use national colours	Keep background theme colourful and eye-catching	Bright colours are use, namely orange and yellow	Bright colours are use, namely orange and yellow	Bright colours are use, namely orange and yellow	Bright colours are use, namely orange and yellow	Bright colours are use, namely orange and yellow
		Add an animation or jingle (e.g., Vietnam) depending on the local context which can be used for continual identification of the brand	The name Taburia is written in colourful letters in a "logo" format	The name Taburia is written in colourful letters in a "logo" format	The name Taburia is written in colourful letters in a "logo" format	The name Taburia is written in colourful letters in a "logo" format	The name Taburia is written in colourful letters in a "logo" format

**Directions for use**	Ideally with pictograms showing steps for the appropriate use of MNP	A pictogram showing steps for the use of MNP is shown (2 images). Image 1 shows Taburia being sprinkled on a meal on a plate. Image 2 shows the meal on a plate.	A more elaborate pictogram showing steps for the appropriate use of MNP is shown (4 images). Image 1 shows a bowl in which 1/3 of the meal is set aside. Image 2 shows Taburia being sprinkled onto 1/3 of the meal. Image 3 shows a spoon stirring Taburia with 1/3 of the meal. Image 4 shows a spoon being used to feed the meal.	*No pictogram*	A pictogram showing steps for the use of MNP is shown (2 images). Image 1 shows Taburia being sprinkled on a meal on a plate. Image 2 shows the meal on a plate.	A more elaborate pictogram showing steps for the appropriate use of MNP is shown (4 images). Image 1 shows a bowl in which 1/3 of the meal is set aside. Image 2 shows Taburia being sprinkled onto 1/3 of the meal. Image 3 shows a spoon stirring Taburia with 1/3 of the meal. Image 4 shows a spoon being used to feed the meal.
		"Take one sachet of Taburia sprinkle it"	*"Shake, tear the sachet and sprinkle all the content..."*	*"Mix contents of one sachet of Taburia …"*	*"Add one sachet …"*	*"Shake, tear the sachet and sprinkle all the content ..."*
		"… on prepared breakfast"	*"Prepare semi solid food/porridge"*	*"…. to the already prepared food of the child …"*	*"… to ready-to-eat solid food (rice, side dishes, porridge, biscuit, fruits, etc.). Give Taburia during breakfast"*	*"Prepare semi solid food/porridge."*
		-	*" … to the 1/3 portion"*	-	-	*"Take 1/3 portion"*
		-	*"Mix well"*	*"Mix contents of Taburia sachet …"*	-	*"Mix well"*
		"Ensure that the food with added Taburia is fully consumed"	*"Ensure that the food with added Taburia is consumed within 30 minutes"*	*"…. make sure it is eaten fully"*	-	*"Ensure that the food with added Taburia is consumed within 30 minutes"*
		-	*"Wash hands with soap before preparing the food and feeding the child"*	-	-	*"Wash hands with soap before preparing the food and feeding the child"*
		"Do not mix with liquid foods and drinks such as soup, milk, or tea to prevent clotting"	*"Do not mix with liquid foods and drinks"*	-	*"Do not mix with liquid foods and drinks such as soup, milk, or tea to prevent clotting"*	*"Do not mix with liquid foods and drinks"*
		"Do not mix with hot foods because some nutrients will be destroyed"	*"Do not mix with hot foods"*	-	*"Do not mix with hot foods because some nutrients will be destroyed"*	*"Do not mix with hot foods"*
		-	-	-	*"Do not share the food that was sprinkled with 1 sachet of Taburia with other family members, because the nutrients are sufficient only for 1 child."*	-

			“Best before ...”	*“Best before ...”*	-	-	-
			“Do not use: If sachet has holes, tears or is deflated”	*“Do not use: If sachet has holes, tears or is deflated”*	*“Do not use: If packaging is damaged”*	-	-
			“Do not use: If the content solidifies, clots or changes colours upon opening”	*“Do not use: If the content solidifies, clots or changes colours upon opening”*	-	*“Prevent packaging from being damaged, torn and deflated, and keep content from clotting and changing colours”* Image of torn box in included.	-
**Frequency of use**	The frequency of recommended use		*“Give Taburia once every 2 days”*	*“Give Taburia once every 2 days”*	-	*“Use one sachet of Taburia once a day for children u5”*	*“Give Taburia once every 2 days with semi solid-food/porridge”*
			“Net weight: 30 sachets @ 1 g”	*“30 × 1 g sachets”*	*“Serving Size 1 sachet (1 g)”*	-	-
			List of ingredients:	List of ingredients:	List of ingredients:		
**Composition**	Specific content formulation in 1 g sachet	*“Contains XX vitamins and minerals” “Provides one daily amount of XX vitamins and minerals” “Contains iron” “Contains XX”*	Nutrient composition per serving (1 g): energy 0 kcal, fat 0 g, protein 0 g, carbohydrates 1 g, sodium 0 mg, and % contribution to “Dietary Reference Intake” for 16 micronutrients (Vitamin A 95%, Vitamin D 100%, Vitamin E 85%, Vitamin K1 110%, Vitamin B1 75%, Vitamin B2 85%, Vitamin B3 75%, Folic acid 80%, Pantothenic acid 100%, Vitamin B6 85%, Vitamin B12 95%, Vitamin C 95%, Iron (Fe) 120%, Iodine 45%, Zinc (Zn) 55% and Selenium (Se) 105%”	Nutrient composition per serving (1 g): energy 0 kcal, fat 0 g, protein 0 g, carbohydrates 1 g, sodium 0 mg, and % contribution to “Dietary Reference Intake” for 16 micronutrients (Vitamin A 95%, Vitamin D 100%, Vitamin E 85%, Vitamin K1 110%, Vitamin B1 75%, Vitamin B2 85%, Vitamin B3 75%, Folic acid 80%, Vitamin B6 85%, Vitamin C 75%, Iron (Fe) 120%, Iodine 45%, Zinc (Zn) 55% and Selenium (Se) 105%”	Nutrient content and % contribution to “Dietary Reference Intake” is provided	-	-
**Expiration date**	The expiration date		The expiration data is stated on the box	The expiration data is stated on the box	The expiration data is stated on the sachet		
**Storage**	Storage instructions		*“Store Taburia in a closed container (box, jar) which is clean, dry. Store in non-humid and safe place (free of ants, bugs and animals)”*	*“Store Taburia in a closed container (box, jar) which is clean and dry. Store in non-humid and safe place (free of ants, bugs and animals)”*	*“Store in a cool and dry place. Do not use if sachet is damaged.”*	*“Always keep in Taburia Box. Store in non-humid place. Keep away from bugs, rats, cockroaches,* etc.*”* Images of cockroaches and rats are included.	*“Store Taburia in a closed container (box, jar) which is clean and dry. Store in non-humid and safe place (free of ants, bugs and animals)”*

			"Keep out of reach of children"	*"Keep out of reach of children"*		*"Keep out of reach of children"*	*"Keep out of reach of children"*
			"Avoid direct exposure to sunlight"	*"Avoid direct exposure to sunlight"*			*"Avoid direct exposure to sunlight"*
**Logos**	Logos of the agencies involved in the distribution (optional)	Gain credible support for product (branding) from official authority, e.g., "approved by Ministry of Health stamp"	MOH Logo is shown and Indonesian Council of Ulama	MOH Logo is shown and Indonesian Council of Ulama	MOH Logo is shown and Indonesian Council of Ulama	MOH Logo is shown	MOH Logo is shown and Indonesian Council of Ulama
**Producer/Importer**	Producer and importer information		Producer information is provided, the produced is PT. Tiga Pliar	Producer information is provided, the produced is PT. Tiga Pliar	Producer information is provided, the produced is PT. Tiga Pliar	-	-
			FDA Registration and MUI (Halal) Number are provided	FDA Registration and MUI (Halal) Number are provided	FDA Registration and MUI (Halal) Number are provided	-	-

[1] HF-TAG (2015). Planning for program implementation of home fortification with micronutrient powders (MNP): a step-by-step manual, Geneva, Home Fortification Technical Advisory Group; [2] GAIN MNP Toolkit (Document for internal use). [3] The original packaging approved by the Ministry of Health (MOH), decree number 2409 and enacted June 2011; [4] The improved packaging approved by the MOH, decree number 51 and enacted October 2016.
